# Supplementary material for: Possibility of Cell Block Specimens from Overnight-Stored Bile for Next-Generation Sequencing of Cholangiocarcinoma
Source: Cells. 2024 May 28;13(11):925. doi: 10.3390/cells13110925 (PMC11172341; doi:10.3390/cells13110925)
Supplement: Supplementary file 1 [file cells-13-00925-s001.zip › cells-2989711-supplementary.pdf]

Table S1. Basic characteristics of cholangiocarcinoma cases.

	n = 42
Age, median (range)	75 (49–98)
Female, n (%)	14 (33)
Etiology	
Extrahepatic cholangiocarcinoma	24
Intrahepatic cholangiocarcinoma	2
Hilar cholangiocarcinoma	8
Gallbladder cancer	8
UICC Stage	
I / II / III / IV	8 / 8 / 12 / 14
Location of stricture, n	
Distal bile duct	27
Hilar bile duct	14
Intrahepatic bile duct	1
Amounts of collected bile (mL), median (range)	180 (80–300)
Malignant cell number of Cell block specimen, median (range) (n=28)	70 (10–2785)
